# Supplementary material for: The effect of deferring feedback on rule-based and information-integration category learning
Source: PLoS One. 2025 Apr 4;20(4):e0313726. doi: 10.1371/journal.pone.0313726 (PMC11970646; doi:10.1371/journal.pone.0313726)
Supplement: Supplementary file 1 [file pone.0313726.s001.odt]

Supplemental Information

# The analyses reported below use the Smith et al. (2014) exclusion criteria for non-learners.

# Experiment 1

In Smith et al. (2014) participants were excluded if they performed significantly lower in the last 100 trials compared with the first 100 trials. Here, we chose to use a chi-squared test as this seemed the most appropriate way to assess this. One participant performed significantly worse in the last 100 trials of the study compared with the first 100 trials and was excluded leaving 85 participants in the following analyses.

	A	2x2	between-subjects	ANOVA	was	conducted	with	category	structure

(UD/II) and feedback type (immediate/deferred) added as the independent variables. The dependent variable was the proportion of correct answers for each participant on the last 100 trials of the study. There was a significant interaction between category structure and feedback type, F(1,81) = 4.26, p = .042, ηg2 = 0.05, BF = 3.41, with deferred feedback significantly impairing accuracy relative to immediate feedback in the II conditions, Mimm = 0.80, SD = 0.12, Mdef = 0.66, SD = 0.10, t(36.4) = 3.99, p <.001, d = 1.24, but not in the UD conditions, Mimm = 0.87, SD = 0.06, Mdef = 0.82, SD = 0.06, t(32.1) = 1.67, p = .11, d = .51. There was also a significant main effect of category structure, F(1,81) = 27.25, p < .001, ηg2 = 0.25, BF = 271744, with participants in the UD conditions, M = 0.84, SD = 0.09, being more accurate than those in the II conditions, M = 0.72, SD = 0.13. In addition, there was a significant main effect of feedback type, F(1,81) = 17.46, p < .001, ηg2 = 0.18, BF  =2213,  where participants in deferred feedback conditions performed less accurately, M = 0.74, SD = 0.13, than participants in immediate feedback conditions, M = 0.83, SD = 0.10.

# Experiment 2a

Four participants were excluded for failing to reach the learning criterion. We conducted a 2 x 2 ANOVA with category structure and feedback type as factors. There was a significant main effect of task, F(1,78) = 46.07, p < .001, ηg2 = 0.37, BF = 26.38, with accuracy higher in the immediate feedback conditions, Mimm = 0.81, SD = 0.1, than the deferred feedback conditions, Mdef = 0.67, SD = 0.09. There was no statistically significant effect of category structure, F(1,78) = 0.52, p = .472, ηg2 = 0.01, BF = .10. The critical interaction between category structure and feedback type was also not significant, F(1,78) = 1.71, p = .195 ηg2 = 0.02, BF = 0.62.

# Experiment 2b

One participant was excluded overall. There was a significant main effect of feedback type, F(1,82) = 24.42,  p < .001, ηg2 = 0.23, BF = 82350, with participants in the immediate condition more accurate, M = 0.79, SD = 0.11, than those in the deferred feedback condition, M = 0.67, SD = 0.12.

	

# Experiment 3a

Three participants were excluded overall. There was  a statistically significant interaction between feedback type and category structure, F(1,80) = 5.80, p = .018, ηg2 = 0.07, BF = 8.72, with deferred feedback impairing II learning, Mimm = 0.80, SD = 0.13, Mdef = 0.63, SD = 0.12, t (40) = 4.37, p<.001, d  = 1.35,  to a greater extent than CJ learning, Mimm = 0.77, SD = 0.08, Mdef = 0.71, SD = 0.08, t(40) = 2.38, p = .022, d = .73. There was also a main effect of feedback condition, F(1,80) = 24.52, p < .001, ηg2 = 0.23, BF = 33924, with participants learning better with immediate feedback, M = 0.78, SD = 0.11, than deferred feedback, M = 0.67, SD = 0.11. The main effect of category structure was not statistically significant, F(1,80) = 1.18, p = .280, ηg2 = 0.01, BF = .21 (II = .71, SD = .15; CJ  = .74 SD = .08).

 	

# Experiment 3b

Three participants were excluded overall. There was a significant interaction between feedback type and category structure, F(1,89) = 4.70, p = .033, ηg2 = 0.05, BF = 6.13, with deferred feedback significantly impairing II learning, Mimm = 0.82, SD = 0.07, Mdef = 0.69, SD = 0.09,  t (42.9) = 5.21), p <.001, d 1.54, but not CJ learning, Mimm = 0.73, SD = 0.10, Mdef = 0.69, SD = 0.10, t(45) = 1.45, p = .15, d = .42. There was a main effect of feedback condition, F(1,89) = 19.45, p < .001, ηg2 = 0.18, BF = 6492, with participants learning better with immediate feedback, M = 0.77, SD = 0.10, than deferred feedback, M = 0.69, SD = 0.10. There was also a significant main effect of category structure, F(1,89) = 6.13, p = .015, ηg2 = 0.06, BF = 13.08, with participants more accurate in the II conditions, M = 0.75, SD = 0.10, than the CJ conditions, M = 0.71, SD = 0.10.
